# Age of Data at the Time of Publication of Contemporary Clinical Trials

**DOI:** 10.1001/jamanetworkopen.2018.1065

**Published:** 2018-08-10

**Authors:** John Welsh, Yuan Lu, Sanket S. Dhruva, Behnood Bikdeli, Nihar R. Desai, Liliya Benchetrit, Chloe O. Zimmerman, Lin Mu, Joseph S. Ross, Harlan M. Krumholz

**Affiliations:** 1 Center for Outcomes Research and Evaluation, Yale-New Haven Hospital, New Haven, Connecticut; 2 Section of Cardiovascular Medicine, Department of Internal Medicine, Yale School of Medicine, New Haven, Connecticut; 3 National Clinician Scholars Program, Department of Internal Medicine, Yale School of Medicine, New Haven, Connecticut; 4 Veterans Affairs Connecticut Healthcare System, West Haven; 5 Cardiovascular Research Foundation, New York, New York; 6 Division of Cardiology, Department of Medicine, Columbia University Medical Center/New York-Presbyterian Hospital, New York; 7 Department of Internal Medicine, Yale School of Medicine, New Haven, Connecticut; 8 Section of General Internal Medicine, Department of Internal Medicine, Yale School of Medicine, New Haven, Connecticut; 9 Department of Health Policy and Management, Yale School of Public Health, New Haven, Connecticut

## Abstract

**Importance:**

As medical knowledge and clinical practice rapidly evolve over time, there is an imperative to publish results of clinical trials in a timely way and reduce unnecessary delays.

**Objectives:**

To characterize the age of clinical trial data at the time of publication in journals with a high impact factor and highlight the time from final data collection to publication.

**Design and Setting:**

A cross-sectional analysis was conducted of all randomized clinical trials published from January 1 through December 31, 2015, in the *Annals of Internal Medicine, BMJ, JAMA, JAMA Internal Medicine, Lancet,* and *New England Journal of Medicine*. Multivariable linear regression analyses were conducted to assess whether data age (adjusted for follow-up duration) and publication time were associated with trial characteristics.

**Main Outcomes and Measures:**

The outcome measures were the midpoint of data collection until publication (data age), the time from first participant enrollment to last participant enrollment (enrollment time), and the time from final data collection to publication (publication time).

**Results:**

There were 341 clinical trials published in 2015 by the 6 journals. For assessment of the primary end point, 37 trials (10.9%) had a follow-up period of less than 1 month, 172 trials (50.4%) had a follow-up period of 1 month to 1 year, and 132 trials (38.7%) had a follow-up period of more than 1 year. For all trials, the median data age at publication was 33.9 months (interquartile range, 23.5-46.3 months). Among trials with a follow-up period of 1 month or less, the median data age was 30.6 months (interquartile range, 18.6-39.0 months). A total of 68 trials (19.9%) required more than 4 years to complete enrollment. The median time from the completion of data collection to publication was 14.8 months (interquartile range, 7.4-22.2 months); publication time was 2 or more years in 63 trials (18.5%). In multivariable regression analyses adjusted for follow-up time, inconclusive or unfavorable trial results were significantly associated with older data age (>235 days). Compared with trials funded only by private industry, trials funded by government were associated with a significantly longer time to publication (>180 days).

**Conclusions and Relevance:**

Clinical trials in journals with a high impact factor were published with a median data age of nearly 3 years. For a substantial proportion of studies, time for enrollment and time from completion of data collection to publication were quite long, indicating marked opportunities for improvement in clinical trials to reduce data age.

## Key Points

**Question:**

What is the age of clinical trial data at the time of publication?

**Findings:**

This cross-sectional analysis of clinical trials published in 2015 in 6 journals with a high impact factor found that by the time of publication, the median data age was nearly 3 years. The median publication time was more than 1.2 years, with 18.5% of trials taking 2 or more years to be published.

**Meaning:**

Collectively, these findings suggest opportunities to adjust various processes related to clinical trials to allow dissemination of the final results in a more timely manner.

## Teaser

This cross-sectional analysis characterizes the age of clinical trial data at the time of publication in journals with a high impact factor and examines the time from final data collection to publication.

## Introduction

Clinical trials require time to generate and disseminate new knowledge. The time lag is subject to fixed constraints, such as the follow-up period for the primary end point, and modifiable factors, such as participant enrollment time and time to publication after completion of data collection. Furthermore, time to publication could be affected by the time needed for data entry, adjudication, cleaning, analysis and interpretation, manuscript preparation, peer reviews, and actual publication by the journal after acceptance. Some of these tasks could be conducted, in large part, in parallel with the conduct of the trial. As medical knowledge and clinical practice rapidly evolve,^1^ the faster the variable aspects of a trial are accomplished, the more relevant the results are to current practice.^2,3^ There is an imperative to publish clinical trial results in a timely way and reduce unnecessary delays.

Previous studies of published clinical trials showed that it took, on average, 2 years for these trials to be published after completion (ie, time to publication).^4,5^ Little is known about the overall age of data at publication, the contribution of the time to publication to the data age, or the time spent enrolling participants. Such information might identify opportunities to accelerate the timeliness of the clinical trial process and reporting. Accordingly, we sought to characterize data age, enrollment time, and publication time of all clinical trials published in 2015 by the medical journals with the highest impact factors.

## Methods

### Data Collection

We screened all original research articles published (either online or in print) from January 1 through December 31, 2015, in 6 general and internal medicine journals with high impact factors^6^: the *New England Journal of Medicine* (*NEJM*), *Lancet, JAMA, BMJ, Annals of Internal Medicine,* and *JAMA Internal Medicine*. We identified only trials with a randomized comparison of an intervention with a control and excluded those that did not represent a primary analysis or that did not report the time of starting enrollment or ending data collection in a specific month (eFigure 1 in the Supplement). Approval of this study was waived by the Yale University institutional review board as it did not meet the definition of human participants research. This study followed the Strengthening the Reporting of Observational Studies in Epidemiology (STROBE) reporting guidelines.

All data elements were independently abstracted by 1 of us (J.W., L.B., C.O.Z., or L.M.) and were then checked for accuracy by a different investigator from our group. For 335 of the 341 data elements (98.2%), the results of both abstractions were in agreement. All discrepancies were resolved through discussion with a third investigator (J.W., J.S.R., or H.M.K.).

### Outcome Measures

**Figure 1.**
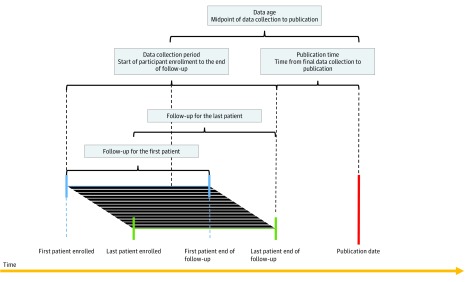
A Mock Trial With Different Time Periods Labeled Progression of a mock trial defining data age, data collection period, and publication time. The black lines indicate an individual patient’s own timeline throughout the study.

The outcome measures of interest were the midpoint of data collection until publication (data age), the time from first participant enrollment to last participant enrollment (enrollment time), and the time from final data collection to publication (publication time). We defined the data collection period as the start of enrollment to the end of follow-up. The definition of data age was determined as such to convey the mean age of the entire sample. The first patient enrolled in the trial could be viewed as the age of the data, as could the last patient follow-up collected. However, using these markers would not capture the sample as well as the midpoint of these 2 time points (Figure 1). We extracted the start and end dates of enrollment, as well as the final data collection date from the main text of each article. When those dates were not available, we checked the trial protocol or appendix and, if necessary, its registration on ClinicalTrials.gov. For trials that provided only a month as a start or end date, we assumed that the start date was the first day of that month and that the end date was the last day of that month. Only 27 of 341 trials (7.9%) were missing the start or end date, and the maximum that a trial duration could have been overestimated would be by 2 months. We extracted the publication date based on the e-publication ahead of print date when it was listed; otherwise, it was based on the print publication date.

### Independent Variables

Our independent variables included important trial features to characterize our sample and features that may be associated with data age at publication. Specifically, we collected data on the type of intervention (drug, device, or other), early study termination for any reason, number of patients enrolled, trial location (United States only, United States and outside of United States, or outside of United States only), number of trial centers, number of manuscript authors, author affiliation (government or private industry), funding source (government, nonprofit, private industry, or combination), whether the trial was registered on ClinicalTrials.gov or registered on another website, and whether results were posted on ClinicalTrials.gov. We also determined the favorability of the findings by designating trials with primary end point results that yielded statistically significant better outcomes for the treatment population compared with the control population as “favorable,” statistically significant worse outcome for the treatment population compared with the control population as “unfavorable,” and all others as “inconclusive.”

### Statistical Analysis

We calculated the median values and interquartile ranges (IQRs) for continuous variables because we presumed the distributions of these variables were not normal; we calculated the total counts and percentages for categorical variables. We reported the data age, enrollment time, and publication time, overall and by follow-up duration (<1 month, 1 month to 1 year, and >1 year). We conducted bivariate analysis to test whether data age, enrollment time, and publication time were associated with each of the following variables: type of intervention, early study termination for any reason, number of patients enrolled, trial location, number of trial centers, number of manuscript authors, author affiliation, funding source, favorability of the findings, trial registration on ClinicalTrials.gov, and results posted on ClinicalTrials.gov. To identify trial characteristics associated with data age, enrollment time, and publication time, we further developed multivariable linear regression models. We adjusted for variables that reached statistical significance at 2-sided *P* < .05 in bivariate analyses. Because longer follow-up duration was associated with older data age of the trials, we also adjusted for follow-up time in the model to account for the different follow-up times in these studies. In a post hoc analysis, we characterized the 10 studies with the longest data age and the 10 studies with the shortest data age. All data were analyzed with R, version 3.3.2 (The R Foundation for Statistical Computing).

## Results

Our search identified 979 original research articles, of which 566 were excluded because they were not randomized clinical trials, 23 because they did not represent a primary analysis, 46 because they had missing data on outcomes of interest, and 3 for other reasons; thus, our final analysis included 341 trials (eFigure 1 in the Supplement). The 341 trials assessed drugs (206 [60.4%]), devices (21 [6.2%]), and other interventions (114 [33.4%]) (Table 1). Among these trials, 37 (10.9%) had a follow-up period of less than 1 month, 172 (50.4%) had a follow-up period between 1 month and 1 year, and 132 (38.7%) had a follow-up period of more than 1 year. The median number of enrollees was 467 (IQR, 212-1260), the median number of trial centers was 23 (IQR, 6-62), and the median number of authors was 16 (IQR, 11-22).

**Table 1.** Characteristics for Randomized Trials Published in 2015 in 6 Journals With High Impact Factors

Characteristic	Trials, No. (%) (N = 341)
Included trial articles by journal	
* Annals of Internal Medicine*	17 (5.0)
* BMJ*	24 (7.0)
* JAMA*	58 (17.0)
* JAMA Internal Medicine*	17 (5.0)
* New England Journal of Medicine*	124 (36.4)
* Lancet*	101 (29.6)
Enrolled patients, median (IQR), No.	467 (212-1260)
Trial type	
Drug	206 (60.4)
Device	21 (6.2)
Other	114 (33.4)
No. of trial centers, median (IQR)	23 (6-62)
Trial location	
United States only	70 (20.5)
United States and outside United States	104 (30.5)
Outside United States only	167 (49.0)
No. of authors, median (IQR)	16 (11-22)
Manuscripts with ≥1 author primarily employed by private industry, No./total No. (%)	
≥1	124/340 (36.5)
0	216/340 (63.5)
Early stoppage of trial	
Yes	21 (6.2)
No	320 (93.8)
Trial registration and results reporting on ClinicalTrials.gov	
Yes	100 (29.3)
No	179 (52.5)
Registered on a site other than ClinicalTrials.gov	60 (17.6)
Unregistered	2 (0.6)
Favorability of findings for the treatment population relative to the control population	
Favorable	231 (67.7)
Unfavorable	12 (3.5)
Inconclusive	96 (28.2)
NA^a^	2 (0.6)
Funding source	
Government	112 (32.8)
Nonprofit	35 (10.3)
Private industry	108 (31.7)
Government, nonprofit	36 (10.6)
Government, private industry	17 (5.0)
Nonprofit, private industry	10 (2.9)
Government, nonprofit, private industry	20 (5.9)
Not disclosed	2 (0.6)
None	1 (0.3)

Abbreviations: IQR, interquartile range; NA, not applicable.

^a^ Two trials had more than 1 primary outcome and had mixed findings.

### Data Age, Enrollment Time, and Publication Time

**Figure 2.**
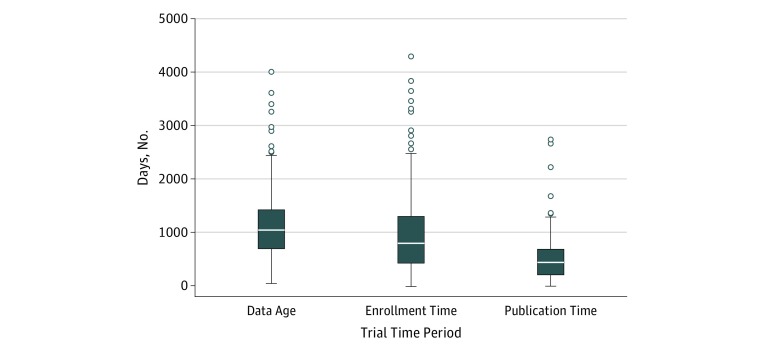
Distributions of Data Age, Enrollment Time, and Publication Time The ends of the boxes indicate the upper and lower quartiles, so the box spans the interquartile range. The middle line indicates the median, the whiskers are the 2 lines outside the box that extend to the highest and lowest observations, and the circles indicate the extreme values of the observations.

The median data age (midpoint of data collection to publication) was 33.9 months (IQR, 23.5-46.3 months; range [minimum to maximum], 2.2-131.8 months). A total of 88 trials (25.8%) reported data age of less than 2 years; 31 trials (9.1%) reported data age of 5 years or more (Figure 2 and eFigure 2 in the Supplement). The median data age was 30.6 months (IQR, 18.6-39.0 months) for trials with a follow-up period of 1 month, 31.8 months (IQR, 21.0-41.7 months) for trials with a follow-up period of 1 month to 1 year, and 40.1 months (IQR, 30.3-51.9 months) for trials with a follow-up period of more than 1 year.

The median enrollment time was 26.2 months (IQR, 14.2-42.3 months; range, 0.3-141.1 months), and the median mean enrollment time per person across trials was 1.4 days (IQR, 0.5-3.8 days; range 0.0002-69.0 days); 64 of 313 trials (20.4%) completed enrollment within 1 year; 251 of 313 trials (80.2%) completed enrollment within 4 years; and 68 trials (19.9%) required more than 4 years to complete enrollment. A total of 257 of 313 trials (82.1%) required fewer than 5 days per enrolled participant; 32 of 313 trials (10.2%) required 9 days or more (Figure 2 and eFigure 3 in the Supplement).

The median time to publication was 14.8 months (IQR, 7.4-22.2 months; range, 0.5-90.3 months) (Figure 2). A total of 138 trials (40.5%) were published within 1 year after completing the final data collection, and 63 trials (18.5%) were published more than 2 years after completing the final data collection (eFigure 4 in the Supplement). Overall, 43.2% (IQR, 26.8%-61.6%) of the data age was the time to publication.

### Factors Associated With Data Age, Enrollment Time, and Publication Time

In multivariable analyses, some factors were associated with older data age, adjusted for follow-up time (Table 2). Compared with favorable trials, inconclusive or unfavorable trials had a median data age that was 235 days longer (95% CI, 108-362 days). Each additional day of follow-up duration was also associated with an additional 0.6 days (95% CI, 0.5-0.8) of data age.

**Table 2.** Factors Associated With Data Age (in Days) in Bivariate and Multivariable Analyses

Factor	Coefficient (95% CI)	
	Bivariate Analysis	Multivariable Analysis^a^
Trial type		
Drug	1 [Reference]	NA
Device	−22 (−238 to 282)	NA
Other	91 (−42 to 223)	NA
No. of enrolled patients (per 1000)	0.1 (−1.3 to 1.5)	NA
No. of trial centers	0.1 (−0.5 to 0.6)	NA
No. of authors	−3.1 (−9.3 to 3.1)	NA
Trial location		
United States only	1 [Reference]	NA
Outside of United States only	86 (−75 to 246)	NA
United States and outside of United States	−116 (−290 to 58)	NA
Manuscripts with ≥1 author primarily employed by private industry		
No	1 [Reference]	NA
Yes	−276 (−401 to −151)^b^	−182 (−376 to 13)
Early stoppage of trial		
No	1 [Reference]	NA
Yes	−47 (−303 to 210)	NA
Trial registration and results reported at ClinicalTrials.gov		
Yes	1 [Reference]	NA
No or unregistered on any site	−30 (−173 to 112)	NA
Registered on a site other than ClinicalTrials.gov	15 (−171 to 200)	NA
Favorability of findings for the treatment population relative to the control population		
Favorable	1 [Reference]	NA
Unfavorable or inconclusive^b^	307 (179 to 436)	235 (108 to 362)
Funding source		
Government	1 [Reference]	NA
Nonprofit	15 (−199 to 229)	76 (−119 to 271)
Private industry	−266 (−415 to −117)^b^	−74 (−297 to 149)
Government and nonprofit	−0.9 (−213 to 211)	46 (−155 to 246)
Private industry and others (government or nonprofit)	267 (30 to 504)^b^	191 (−43 to 425)
Government, nonprofit, and private industry	−17 (−251 to 286)	219 (−49 to 487)
Follow-up duration (per day)^b^	0.6 (0.4 to 0.7)	0.6 (0.5 to 0.8)

Abbreviation: NA, not applicable.

^a^ We selected variables that were significant in the bivariate analysis and included them in the multivariable analysis. We also adjusted for follow-up time in the multivariable analysis to account for the different follow-up time in these studies.

^b^ Estimates were statistically significant at *P* < .05.

We also found several characteristics associated with significantly longer enrollment time (Table 3). Specifically, trials that had no authors affiliated with private industry were associated with a longer enrollment time (by 566 days; 95% CI, 306-827 days) than those with at least 1 author affiliated with industry. Compared with trials that had only government funding, trials that had funding from both private industry and government or from both private industry and nonprofit agencies (by 460 days; 95% CI, 152-768 days) or all 3 sources together (by 676 days; 95% CI, 322-1031 days) were also associated with a longer enrollment time. Compared with trials that were funded only by private industry, trials that were only government funded were associated with an additional 180 days (95% CI, 18-343 days) to publish (Table 4).

**Table 3.** Factors Associated With Enrollment Time (in Days) in Bivariate and Multivariable Analyses

Factor	Coefficient (95% CI)	
	Bivariate Analysis	Multivariable Analysis^a^
Trial type		
Drug	1 [Reference]	NA
Device	170 (−155 to 496)	NA
Other	63 (−108 to 235)	NA
No. of enrolled patients (per 1000)	−1.1 (−1.1 to 0.9)	NA
No. of trial centers	0.4 (−0.3 to 1.0)	NA
No. of authors	2.1 (−5.9 to 10)	NA
Trial location		
United States only	1 [Reference]	NA
Outside of United States only	148 (−62 to 358)	NA
United States and outside of United States	−91 (−320 to 138)	NA
Manuscripts with ≥1 author primarily employed by private industry		
No	1 [Reference]	NA
Yes^b^	−457 (−618 to −297)	−566 (−827 to −306)
Early stoppage of trial		
No	1 [Reference]	NA
Yes	35 (−285 to 355)	NA
Trial registration and results reported at ClinicalTrials.gov		
Yes	1 [Reference]	NA
No or unregistered on any site	71 (−113 to 255)	NA
Registered on a site other than ClinicalTrials.gov	3.1 (−236 to 242)	NA
Funding source		
Government	1 [Reference]	NA
Nonprofit	36 (−99 to 171)	−23 (−283 to 236)
Private industry	−157 (−251 to −63)^b^	156 (−136 to 448)
Government and nonprofit	−67 (−200 to 67)	129 (−138 to 395)
Private industry and others (government or nonprofit)	−17 (−166 to 132)	460 (152 to 768)^b^
Government, nonprofit, and private industry	−175 (−344 to −6)	676 (322 to 1031)^b^

Abbreviation: NA, not applicable.

^a^ Only variables significant in the bivariate analysis were included in the multivariable analysis.

^b^ Estimates were statistically significant at *P* < .05.

**Table 4.** Factors Associated With Publication Time (in Days) in Bivariate and Multivariable Analyses

Factor	Coefficient (95% CI)	
	Bivariate Analysis	Multivariate Analysis^a^
Trial type		
Drug	1 [Reference]	NA
Device	−22 (−183 to 139)	NA
Other	76 (−5.9 to 158)	NA
No. of enrolled patients (per 1000)	0.6 (−0.3 to 1.4)	NA
No. of trial centers^b^	−0.5 (−0.8 to −0.2)	−0.4 (−0.7 to −0.1)
No. of authors^b^	−6.6 (−10.4 to −2.8)	−5.9 (−9.9 to −2.0)
Trial location		
United States only	1 [Reference]	NA
Outside of United States only	11 (−88 to 111)	62.3 (−42 to 167)
United States and outside of United States	−123 (−231 to −16)^b^	30 (−104 to 164)
Manuscripts with ≥1 author primarily employed by private industry		
No	1 [Reference]	NA
Yes	−88 (−167 to −9)^b^	98 (−35 to 231)
Early stoppage of trial		
No	1 [Reference]	NA
Yes	−115 (−274 to 44)	NA
Trial registration and results reported at ClinicalTrials.gov		
Yes	1 [Reference]	NA
No or unregistered on any site	−19 (−107 to 69)	NA
Registered on a site other than ClinicalTrials.gov	70 (−45 to 185)	NA
Favorability of findings for the treatment population relative to the control population		
Favorable	1 [Reference]	NA
Unfavorable or inconclusive	93 (11 to 175)^b^	38 (−51 to 128)
Funding source		
Government	1 [Reference]	NA
Nonprofit	36 (−98 to 171)	−4.5 (−145 to 136)
Private industry^b^	−157 (−251 to −63)	−180 (−343 to −18)
Government and nonprofit	−67 (−200 to 67)	−63 (−201 to 75)
Private industry and others (government or nonprofit)	−17 (−166 to 133)	−27 (−184 to 131)
Government, nonprofit, and private industry	−175 (−344 to −6.3)^b^	−167 (−351 to 17)

Abbreviation: NA, not applicable.

^a^ Only variables significant in the bivariate analysis were included in the multivariable analysis.

^b^ Estimates were statistically significant at *P* < .05.

In a post hoc analysis, we characterized 10 studies with the longest data age. They were mostly drug trials (7), were mostly non-US based (6), and had an end of follow-up to publication time range of 7.8 to 91.5 months. In contrast, the 10 studies with the shortest data age shared the following characteristics: 9 were drug trials, all had no follow-up or relatively short follow-up, the end of follow-up to publication time range was 0.5 to 6 months, they involved higher numbers of trial centers, and the area of study was predominately of the hepatitis C virus or Ebola.

## Discussion

In our review of clinical trials published in 2015 in 6 journals with high impact factors, we found that by the time of publication, the median data age was nearly 3 years and the median publication time was more than 1.2 years, with 63 trials (18.5%) taking 2 years or more to complete. For some trials, enrollment periods required as many as 9 days per participant. In multivariable analyses, inconclusive or unfavorable trial results (vs favorable results) were significantly associated with older data age after adjusting for follow-up time. Government-funded trials took 6 months longer in time to publication. Collectively, these findings suggest opportunities to adjust various processes related to clinical trials to improve the timeliness for dissemination of the final results.

Our study extends the current literature in 2 important ways. First, previous studies have shown delays in publication, which we confirm with a comprehensive assessment, in addition to elucidating data age and enrollment time as important time markers of clinical trials.^4,5^ Our descriptive analysis of the data age, enrollment time, and time to publication of randomized trials in medical journals with the highest impact factors provides benchmarks and indicates leverage points to improve the timeliness for research dissemination. As medical knowledge rapidly evolves, an old data age and a long delay in publication time can result in the knowledge generated from trials being less relevant to contemporary clinical practice.^7,8,9^ In addition, there could be implications for research—researchers trying to apply the trial findings and advance the science will be delayed in adapting the new knowledge.

Our study also adds to the literature on which trial characteristics are associated with the time needed for each phase of a trial from the start of enrollment to the dissemination of results to other investigators, clinicians, policy makers, and patients. We found that trials with more centers and more authors were significantly associated with shorter times to publication, but the effect sizes were small. Of greater importance, government funding was associated with substantially longer times to publication. Researchers funded by private companies may have shorter publication times because of greater incentives to produce and distribute findings compared with researchers funded by government grants or nonprofit foundations. Private funders may impose greater accountability on the clinical trial process to match the performance of industry; they may also provide more resources, better staffing, larger infrastructure, and share knowledge of patent drugs, devices, or other interventions to improve timeliness. There are many other factors that may be responsible for the differences between trials funded by private industry and those funded by the government, including resources, the use of contract research organizations, and motivation. The ultimate goal is to identify best practices and spread them. For some trials, particularly in the area of prevention, which depend on the accumulation of the hard end points over time, a longer follow-up time is required, and it is inevitable that these trials will have older data age at the time of publication. However, our study shows that, aside from the follow-up time, multiple areas contribute to older data age, including enrollment and publication times. Our findings reveal many opportunities in these areas where the clinical trial process can be accelerated and the time from data collection to publication (ie, the data age) can be shortened.

First, because there is variability in enrollment rates, the trials with relatively slow enrollment (>9 days per participant) may need to consider innovative strategies. Enrollment time might be shortened by integrating the randomization process into clinical practice, such as by the use of already existing clinical registries.^10^ Examples include the SAFE-PCI for Women (Study of Access Site for Enhancement of PCI for Women) trial^11^ using the National Cardiovascular Research Infrastructure as the platform for randomization and data collection, and the ADAPTABLE (Aspirin Dosing: A Patient-Centric Trial Assessing Benefits and Long-term Effectiveness) trial^12^ using the National Patient-Centered Clinical Research Network to support rapid and efficient randomization of patients. In this way, participants could be enrolled (and data generated) more quickly. Another approach might be preregistration of participants (ie, creating a pool of people who are amenable to enrollment in trials), so that such individuals are easier to identify and invite to participate. It may also be useful to make enrollment less reliant on clinicians and pursue more direct-to-participant strategies. The idea of participant-partnered research is growing and could provide opportunities to disrupt the current approaches.^13,14^

Another opportunity for improvement is publication time, which might be shortened by accelerating the aggregation and analysis of data, the time to write manuscripts, and the submission and revision processes before publication. Of the 6 journals that published the work examined in this study, only *BMJ* publicly provides this information, and it shows that the peer review process contributes to a substantial proportion of the publication time. It is possible that some manuscripts may be reviewed and rejected at other journals before they are accepted by a different journal, which could lead to some delays. An implication of this study is that there may be more a priori preparation for completion of a manuscript, even before the final data are analyzed. Authors can be preparing the Introduction and Methods of the manuscript even before final results are known, and discussions with journal editors may proceed before the calculation of results. In the end, there needs to be an imperative to report the results of a completed trial quickly and comprehensively. Many recent trials, including the recently published CANTOS (Canakinumab Anti-inflammatory Thrombosis Outcomes Study) trial, demonstrated that a complex trial could have a very short time from completion to publication (58 days).^15^

### Limitations

Our study has several limitations. First, this study, by design, focuses on trials from 6 general medicine journals with the highest impact factors in 1 year, not a complete sample of trials published across all general medicine journals. We selected these journals because of their prominence and because they publish the trials that are likely to inform clinical practice, and thus they should be the best examples for how quickly trials are conducted and published. Because the general medical journals with high impact factors analyzed in this study are more likely to publish more quickly compared with other general medical journals, we may expect longer delays if we include all general medicine journals. Second, we used the midpoint of data collection until publication as the definition of data age, but data might not be collected evenly over time. Therefore, our results may not precisely reflect the true data age, but we expect this difference to be small. Third, we do not have information about duration of the peer review process except for 1 journal; therefore, we are unable to determine whether longer times to publication were caused by submission delays or by the time required for peer review, acceptance, and publication. Fourth, this study cannot determine the effect of older data age in some trials. However, practice is changing rapidly, and it could be that the clinical care patterns at the end of the trial were different than at the beginning. Such interactions of effect are rarely tested. Also, all things being equal, more recent data and a more quickly completed study are preferable. Therefore, data age does seem to be a relevant metric worthy of more attention and study. This study also did not evaluate delays in the translation of the trial findings into practice. Often, even well-done trials experience delays after publication. This issue—which was beyond the scope of our present article—also deserves attention, along with more timely knowledge generation in the course of trials.

## Conclusions

Clinical trials in 6 journals with high impact factors were published with a median data age of nearly 3 years. For a substantial proportion of these trials, there were extended times for enrollment and publication that led to markedly older data at the time of publication. There are seemingly many opportunities for improvement in the clinical trial process and in the work of trialists with journal editors.

## Notes

Supplement. **eFigure 1.** PRISMA Flowchart **eFigure 2.** Distribution of Data Age for Randomized Trials Published in 6 High-Impact Journals in 2015 **eFigure 3.** Distribution of Enrollment Time for Randomized Trials Published in 6 High-Impact Journals in 2015 **eFigure 4.** Distribution of Publication Time for Randomized Trials Published in 6 High-Impact Journals in 2015
