# Supplementary material for: Age of Data at the Time of Publication of Contemporary Clinical Trials
Source: JAMA Netw Open. 2018 Aug 10;1(4):e181065. doi: 10.1001/jamanetworkopen.2018.1065 (PMC6324269; doi:10.1001/jamanetworkopen.2018.1065)
Supplement: Supplement. — eFigure 1. PRISMA Flowchart eFigure 2. Distribution of Data Age for Randomized Trials Published in 6 High-Impact Journals in 2015 eFigure 3. Distribution of Enrollment Time for Randomized Trials Published in 6 High-Impact Journals in 2015 eFigure 4. Distribution of Publication Time for Randomized Trials Published in 6 High-Impact Journals in 2015 [file jamanetwopen-1-e181065-s001.pdf]

Supplementary Online Content

Welsh J, Lu Y, Dhruva SS, et al. Age of data at the time of publication of contemporary clinical trials. *JAMA Netw Open.* 2018;1(4):e181065. doi:10.1001/jamanetworkopen.2018.1065

eFigure 1. PRISMA Flow Chart

eFigure 2. Distribution of Data Age for Randomized Trials Published in 6 High-Impact Journals in 2015

eFigure 3. Distribution of Enrollment Time for Randomized Trials Published in 6 High-Impact Journals in 2015

eFigure 4. Distribution of Publication Time for Randomized Trials Published in 6 High-Impact Journals in 2015

This supplementary material has been provided by the authors to give readers additional information about their work.

© 2018 Welsh J et al. *JAMA Network Open.*

eFigure 1. PRISMA Flow Chart

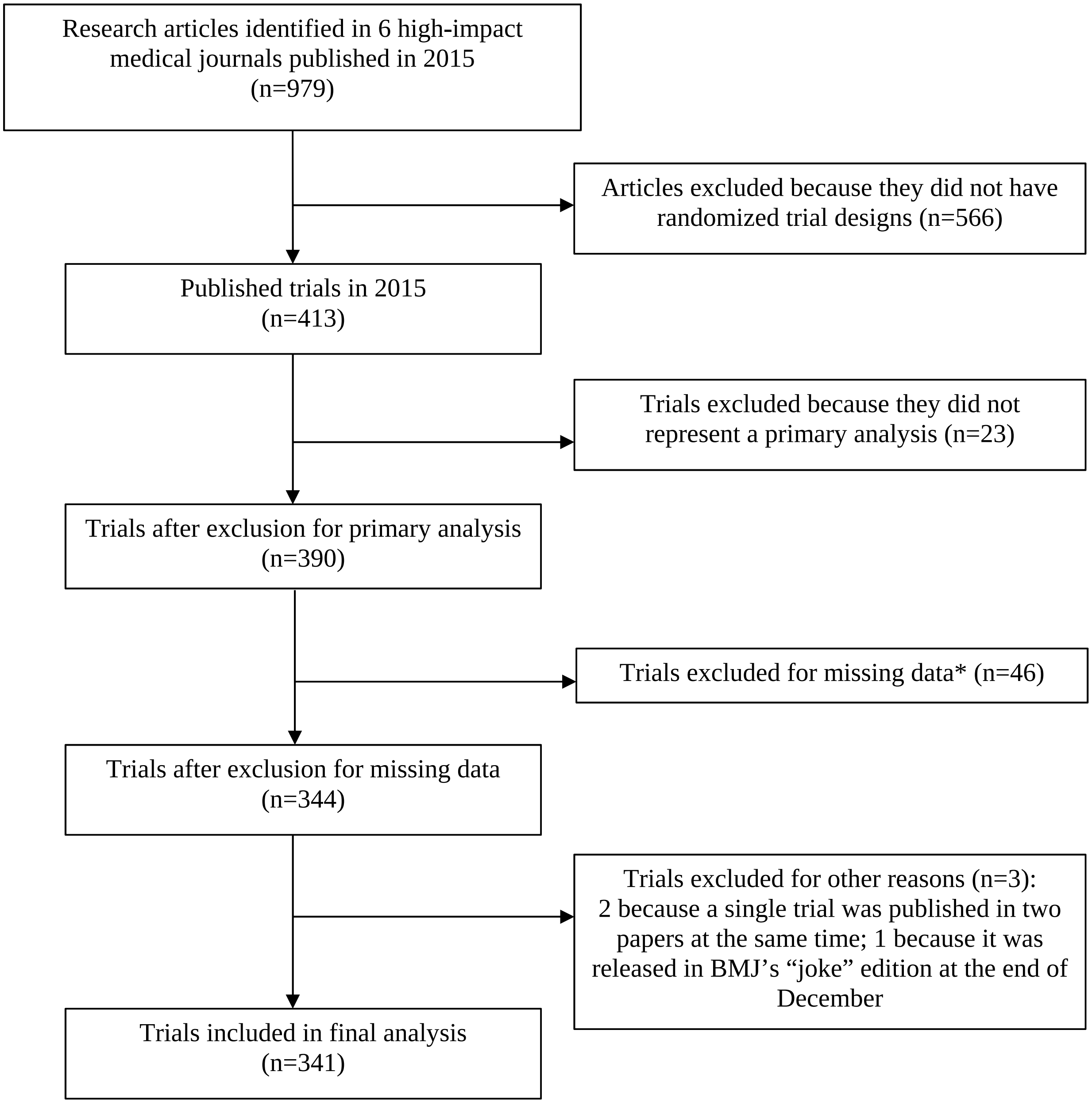

* Missing data included missing date for starting enrollment or ending data collection.

© 2018 Welsh J et al. *JAMA Network Open.*

eFigure 2. Distribution of Data Age for Randomized Trials Published in 6 High-Impact Journals in 2015

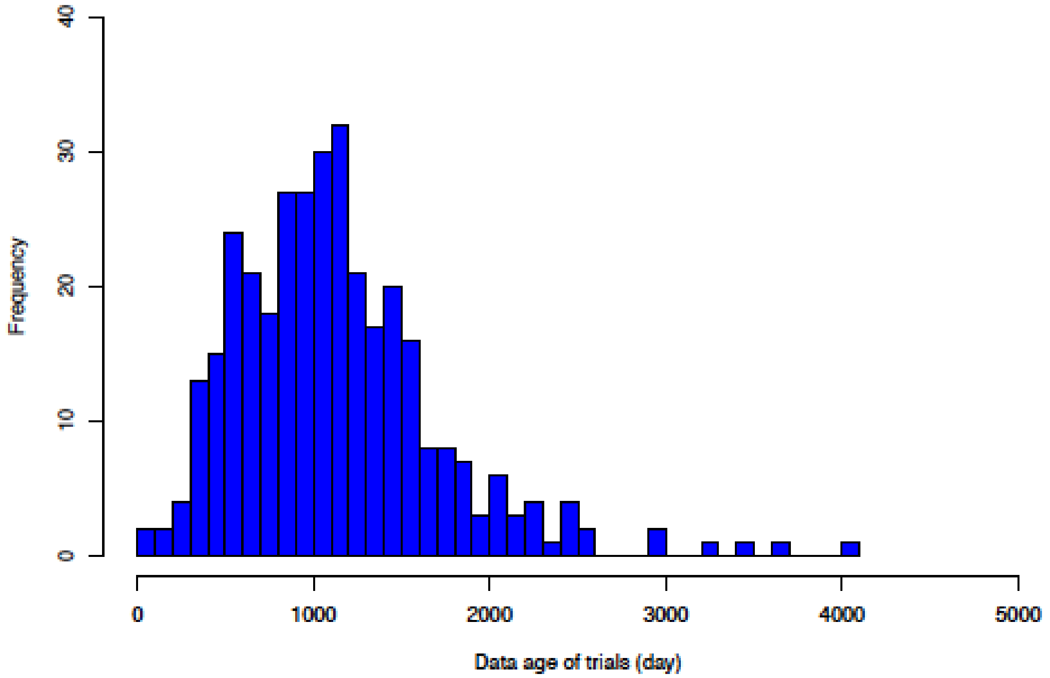

© 2018 Welsh J et al. *JAMA Network Open.*

eFigure 3. Distribution of Enrollment Time for Randomized Trials Published in 6 High-Impact Journals in 2015

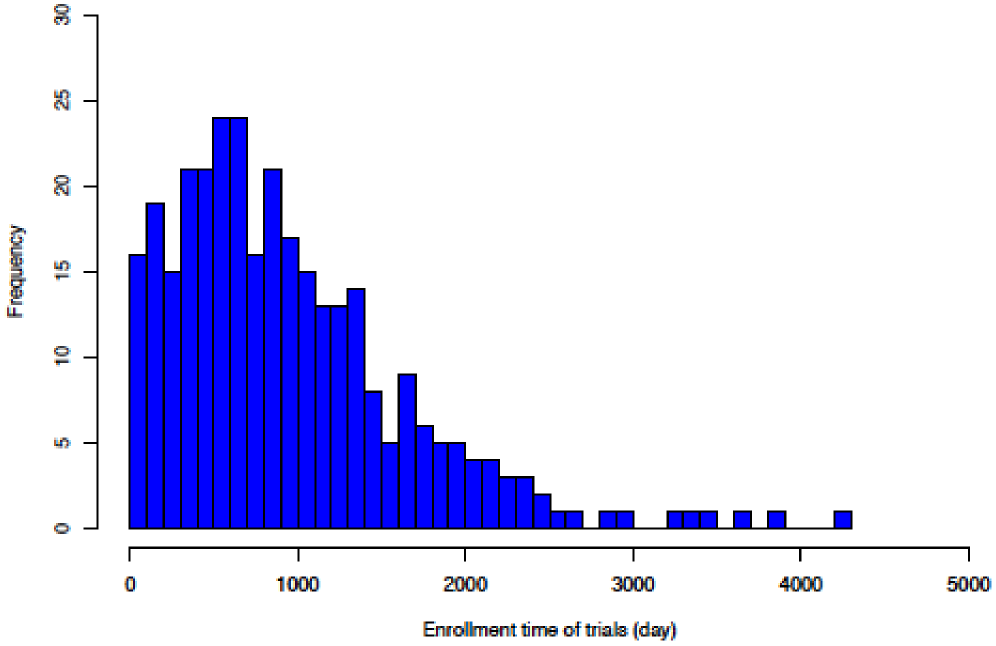

© 2018 Welsh J et al. *JAMA Network Open.*

eFigure 4. Distribution of Publication Time for Randomized Trials Published in 6 High-Impact Journals in 2015

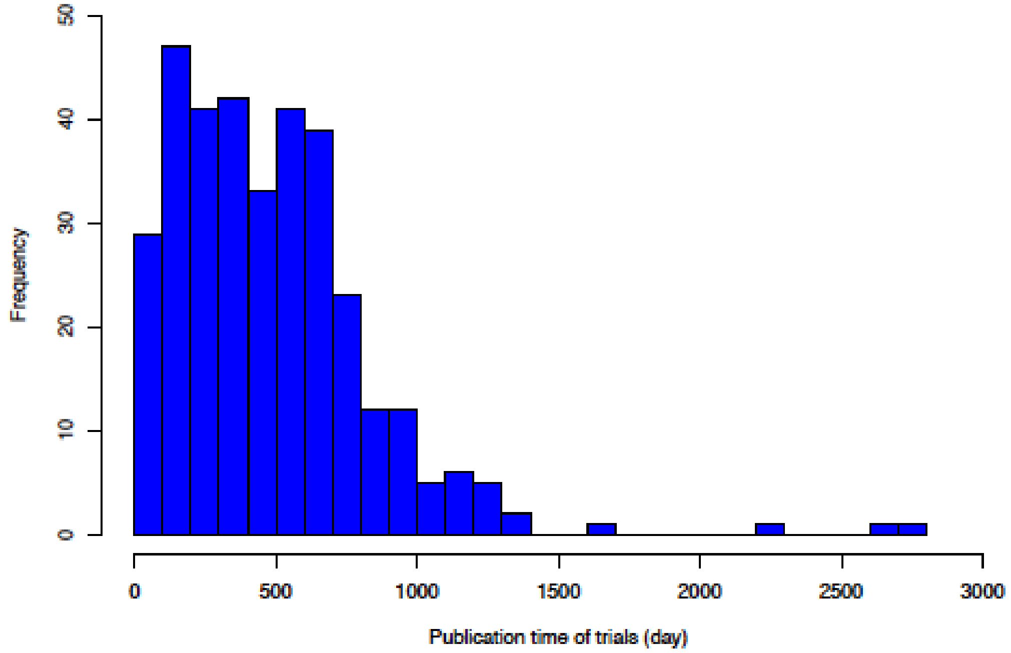

© 2018 Welsh J et al. *JAMA Network Open.*
